# Comparison of quantitative muscle ultrasound and whole-body muscle MRI in facioscapulohumeral muscular dystrophy type 1 patients

**DOI:** 10.1007/s10072-023-06842-5

**Published:** 2023-06-14

**Authors:** Laura Fionda, Fiammetta Vanoli, Antonella Di Pasquale, Luca Leonardi, Stefania Morino, Gioia Merlonghi, Antonio Lauletta, Girolamo Alfieri, Rocco Costanzo, Laura Tufano, Elena Rossini, Elisabetta Bucci, Andrea Grossi, Rossella Tupler, Marco Salvetti, Matteo Garibaldi, Giovanni Antonini

**Affiliations:** 1 Neuromuscular and Rare Disease Centre, Department of Neuroscience, Mental Health and Sensory Organs (NESMOS), Faculty of Medicine and Psychology, SAPIENZA University of Rome, Sant’Andrea Hospital, Via di Grottarossa 1035-1039, 00189 Rome, Italy; 2 Neurology IV-Neuroimmunology and Neuromuscular Diseases Unit, Fondazione IRCCS Istituto Neurologico Carlo Besta, 20133 Milan, Italy; 3 Department of Neurology, E. Agnelli Hospital, Pinerolo, Turin, Italy; 4 Department of Life Sciences, University of Modena and Reggio Emilia, Modena, Italy

**Keywords:** Facioscapulohumeral muscular dystrophy, Muscle disorders, Muscle ultrasound, MRI, Biomarkers

## Abstract

**Introduction:**

Muscle ultrasound is a fast, non-invasive and cost-effective examination that can identify structural muscular changes by assessing muscle thickness and echointensity (EI) with a quantitative analysis (QMUS). To assess applicability and repeatability of QMUS, we evaluated patients with genetically confirmed facioscapulohumeral muscular dystrophy type 1 (FSHD1), comparing their muscle ultrasound characteristics with healthy controls and with those detected by MRI. We also evaluated relationships between QMUS and demographic and clinical characteristics.

**Materials and methods:**

Thirteen patients were included in the study. Clinical assessment included MRC sum score, FSHD score and The Comprehensive Clinical Evaluation Form (CCEF). QMUS was performed with a linear transducer scanning bilaterally *pectoralis major*, *deltoid*, *rectus femoris*, *tibialis anterior* and *semimembranosus muscles* in patients and healthy subjects. For each muscle, we acquired three images, which were analysed calculating muscle EI by computer-assisted grey-scale analysis. QMUS analysis was compared with semiquantitative 1.5 T muscle MRI scale.

**Results:**

All muscles in FSHD patients showed a significant increased echogenicity compared to the homologous muscles in healthy subjects. Older subjects and patients with higher FSHD score presented increased muscle EI. *Tibialis anterior* MRC showed a significant inverse correlation with EI. Higher median EI was found in muscles with more severe MRI fat replacement.

**Conclusions:**

QMUS allows quantitative evaluation of muscle echogenicity, displaying a tight correlation with muscular alterations, clinical and MRI data. Although a confirmation on larger sample is needed, our research suggests a possible future application of QMUS in diagnosis and management of muscular disorders.

## Introduction

Muscular imaging represents an important diagnostic tool for the identification and quantification of muscular alterations in patients affected by neuromuscular disorders (NMDs) [1]. It also represents a useful biomarker for disease progression and treatment effect.

To date, muscle MRI is the most used imaging technique in muscle disorders, thanks to its ability to detect fat infiltration and muscle edema and quantify tissue changes over time [2, 3]. Moreover, it is widely accepted that specific MRI patterns of muscle involvement can be useful in phenotype definition and diagnosis orientation in muscular disorders. Muscle MRI has been also used to grade the severity of muscle involvement by a semiquantitative [4] or a quantitative scale [5]. However, high costs, relative availability of the technique, long exam duration (especially in case of claustrophobia), and the need of sedation in young children represent the most important potential limits of a routine use of muscle MRI.

Muscle ultrasound (US) is a fast, non-invasive, patient friendly and cost-effective examination, which can also be performed at bedside [6]. It is able to identify structural muscular changes by assessing muscle thickness and echointensity (EI), which increases in case of muscle edema, fat infiltration and fibrosis [7, 8]. Muscle US can be performed both by qualitative and quantitative methods. Though the first modality is suitable for a routine examination, quantitative muscle US (QMUS) guarantees a lower operator-dependency and provides more suitable results for statistical analysis and research [9, 10]. In particular, QMUS allows to measure muscle EI by computer-assisted grey-scale analysis, which offers higher sensitivity and reliability compared to visual evaluation in muscle imaging in numerous NMDs [6, 11]. However, although some papers reported QMUS application since 1980s for diagnosing children and adults with NMDs [12], QMUS techniques are not yet standardized, and measurements are not well defined.

To assess applicability and repeatability of QMUS, we compared the muscle US characteristics of a group of patients with genetically confirmed facioscapulohumeral muscular dystrophy type 1 (FSHD1) with those of a matching healthy control group. Muscle US findings of FSHD1 patients were also compared to their muscle MRI characteristics. In addition, we evaluated the correlation between QMUS and demographic and clinical characteristics.

We conducted our study on FSHD1 patients as this is a common muscular dystrophy [13] characterized by a replicable phenotype [14, 15], with a specific and progressive muscle MRI pattern of fat replacement and muscular edema [16, 17].

## Methods

### Patients

We included in this study 13 genetically confirmed FSHD type 1 (FSHD1) adult patients, aged 18 years or over, attending the outpatient service of the neuromuscular clinic at Sant’Andrea Hospital, and 8 healthy controls, recruited from our work environment, matched by sex, age and BMI. Obese subjects were excluded from the study due to excessive attenuation of the ultrasound beam caused by the great thickness of the subcutaneous fat layer. Each patient underwent a neurological examination and QMUS study, which were performed by two different evaluators blinded to each other and to MRI data. The study was conducted according to the principles of the Declaration of Helsinki (version October 2013) and in accordance with the Medical Research Involving Human Subjects Act (WMO). Our institutional ethics committee approved the study and all patients and healthy controls provided written informed consent.

### Clinical evaluation

Each patient underwent a neurological examination, comprehensive of muscle strength evaluation with the Medical Research Council (MRC) scale [18]. For statistical analysis, we considered the MRC score of the *tibialis anterior*, as its function is scarcely influenced by the activity of additional muscles, unlike the other muscles that were included in the study. The Comprehensive Clinical Evaluation Form (CCEF) proposed by the Italian Clinical Network for FSHD [19] and the FSHD clinical score [20] were used to define the clinical category and severity.

### Quantitative muscle ultrasound (QMUS)

Muscle US was performed using a General Electric Voluson E6 imaging system (GE Healthcare, Waukesha, WI) with a broadband linear transducer (frequency band 10–18 MHz), in the same date ± 1 day of clinical assessment and ± 1 month by MRI study by a trained investigator. All muscles were scanned keeping gain, focus, depth and compression constant, to ensure compatibility between each measurement. A generous amount of contact gel was used to reduce the required pressure of the transducer on the muscle. Patients were analysed in supine and prone position, with arms and legs extended and relaxed muscles. The transducer was placed perpendicularly to the muscle and oblique scanning was avoided by changing the angle of the transducer to achieve the best bone EI. QMUS measurements were performed bilaterally on five different muscles, selected because frequently affected (*pectoralis major*, *rectus femoris*, *tibialis anterior and semimembranosus*) or spared (*deltoid*) in FSHD1 and suited for US measurements. Each muscle was scanned in the transverse plane in a specific standard transducer location, which corresponded to the largest muscle diameter at the following sites: *deltoid* about three centimetres above the origin from the acromion process; *pectoralis major* at his humeral head just medially to the axilla; *rectus femoris* halfway along the line from the anterior–superior iliac spine to the superior aspect of the rotula; *tibialis anterior* at one-quarter of the distance from the inferior aspect of the rotula to the lateral malleolus; *semimembranosus* halfway between ischial tuberosity and popliteal fossa. For each muscle, three consecutive screen images were taken and analysed to reduce intra-observer variation in determination of EI. During examination, particular care was taken to keep the transducer in the same exact location and the same standardized position of the patient. Every screen image was then stored offline in DICOM format and analysed using Adobe Photoshop (Adobe System Inc, San Jose, CA, USA). In each stored image, we manually selected a region of interest (ROI) using the calliper function, to include as much muscle mass as possible, excluding the bone and possibly the muscular fascia, which have high EI. The standard histogram function was used to translate the median EI of the ROI into a numeric value between 0 (= black) and 255 (= white) [10, 21]. Quantitative evaluation of all measurements was performed by a technician, blind both to clinical and MRI data.

The whole protocol required about 30 min to acquire the measurements and another 20 min for EI analysis.

### Muscle magnetic resonance

Muscle MRIs were obtained in all patients using a 1.5-T MRI following previously described protocols in accordance with the international consensus recommendations [22]. Patients were placed on their backs with their arms extended at the side. Our muscle MRI protocol includes the evaluation of 39 muscles of the lower body, including pelvic girdle and lower limb muscles, and 18 muscles of the upper body, including scapular girdle and arms, analysing T1 turbo spin echo (T1-TSE) and T2-short tau inversion recovery (T2-STIR) sequences. For the aim of this study, we measured fat replacement on T1 sequences using a 4-point scale (1–4) according to Mercuri classification [23] and edema/inflammation on STIR sequences using a 2-point scale (0: negative, 1: positive) in the muscles, which had been scanned by US.

Two independent neurologists with experience in the analysis of MRIs (LF, MG) blinded to clinical and US features analysed all MRIs. In case of discordance in MRI evaluation, examiners reviewed together the MRI to find an agreement on the final score.

### Statistical analysis

For the statistical analysis, we compared the US quantitative characteristics of each scanned muscle (i.e., *deltoid*, *pectoralis major*, *rectus femoris*, *tibialis anterior and semimembranosus* on both sides) between patients and controls. Median EI in homologous muscles in patients and controls was compared with the Mann–Whitney test. Test–retest reliability was checked to evaluate intraclass correlation coefficient (ICC). Differences in the distribution of median EI in *tibialis anterior* of patients with respect to its strength as evaluated in 3 MRC subcategories (MRC 0–3; MRC 4 and MRC 5) were evaluated with Kruskal–Wallis test.

Linear regression analysis was performed between EI and FSHD score, while the relationship between EI and CCEF was not analysed because of the descriptive nature of this last scale.

A multiple regression analysis was run to predict EI from gender, age and FSHD score.

The difference in the distribution of median EI score between muscles with and without MRI changes (fat replacement and/or edema) was evaluated with Mann–Whitney *U* test. A Kruskal–Wallis test was used to determine if there were differences in the distribution of median EI with respect to the severity of fat replacement as evaluated by the Mercuri scale, considering 3 different sub-categories: no replacement, mild replacement (Mercuri 1–2) and marked replacement (Mercuri 3–4).

## Results

### Patients

Thirteen patients (9 males and 4 females) with genetically confirmed FSHD1 with median age 51 years (IQR: 33–59) and mean body mass index (BMI) 24.50 (*SD*: 3.47) and 8 healthy subjects (5 males and 3 females), with median age of 49.5 years (*IQR*: 31.5–58.75) and mean BMI of 24.50 (*SD*: 2.77), were included in this study. Age difference between patients and controls ranged between 0 and 3 years (mean = 1.50, *SD* = 1.19). BMI difference ranged between 0 and 2.85 kg/mq (mean = 1.40, *SD* = 1.05). A total of 210 muscles were scanned both in patients (130 scanned muscles) and healthy controls (80 scanned muscles)*.*

According to CCEF, 7 patients were assigned to category A2, 2 patients to category A3, 2 patients to category B1 and 1 patient to category A1.

FSHD score ranged from 1 to 8, median 6, *IQR*: 4–7.5 (Table 1).

**Table 1:** Demographic and clinical characteristics of 13 FSHD1 patients

*Patient number*	*Gender*	*Age (yrs.)*	*Allele length (KB)*	*CCEF category*	*FSHD score*
*1*	Male	33	24	A1	5
*2*	Female	33	30	A2	8
*3*	Male	70	24	A2	6
*4*	Male	53	28	A2	6
*5*	Female	51	23	A2	8
*6*	Female	31	24	A1	5
*7*	Male	53	39	A2	3
*8*	Male	56	33	A3	8
*9*	Male	48	25	A2	7
*10*	Male	33	30	B1	1
*11*	Male	33	30	B1	1
*12*	Female	63	25	A2	7
*13*	Male	62	33	A3	6

### QMUS in patients vs. healthy subjects

All muscles in FSHD patients showed a significant increase in EI compared to the homologous muscles in healthy subjects (*deltoid p* < 0.02, right *semimembranous p* = 0.028, all other muscles *p* < 0.001).

Test/retest analysis, both in patients and controls, showed a high reliability in the three determinations (*ICC* = 0.977 and *ICC* = 0.963 respectively). The muscle with the highest median EI was pectoralis major, followed by tibialis anterior, rectus femoris, deltoid and semimembranosus. Values of single muscle median EI in patients and controls are reported in Table 2.

**Table 2:** Values of muscle echointensity in patients and controls

*Muscle*	*Patients*			*Controls*	*p*
	Median	IQR	Median	IQR	
*Pectoralis major R*	91.00	78.00–113.00	55.00	52.25–63.75	< 0.001
*Pectoralis major L*	91.00	72.00–106.00	48.50	40.25–59.75	< 0.001
*Deltoid R*	66.00	52.00–79.00	53.50	52.00–60.50	0.012
*Deltoid L*	57.00	43.00–67.00	46.50	37.50–58.00	0.018
*Rectus femoris R*	73.00	64.00–99.00	52.50	39.00–72.00	< 0.001
*Rectus femoris L*	69.00	54.00–94.00	48.00	42.00–64.50	< 0.001
*Semimembranosus R*	57.00	37.00–87.00	42.50	36.00–47.75	0.028
*Semimembranosus L*	63.00	47.00–86.00	40.00	34.75–42.75	< 0.001
*Tibialis anterior R*	77.00	55.00–102.00	50.00	45.25–60.00	< 0.001
*Tibialis anterior L*	91.00	67.00–111.00	56.00	47.00–65.00	< 0.001

### Correlations of QMUS with demographic and clinical characteristics

Both in patients and controls, EI was linearly correlated with age. EI was higher in female patients and in male healthy controls. EI was directly correlated with FSHD score (*p* < 0.001). There were no correlations between EI and genetic characteristic in terms of allele length (*p* = 0.27). Multiple regression analysis showed that, keeping all other independent variables constant, median EI in FSHD muscles increased overall of 3.41 points for every 10-year increase of age (95% CI: 1.13–5.70; *p* = 0.003) and of 3.47 points for every point of increase in the FSHD score (95% CI: 2.07–4.87; *p* < 0.0001). Gender did not significantly influence the model.

**Fig. 1:**
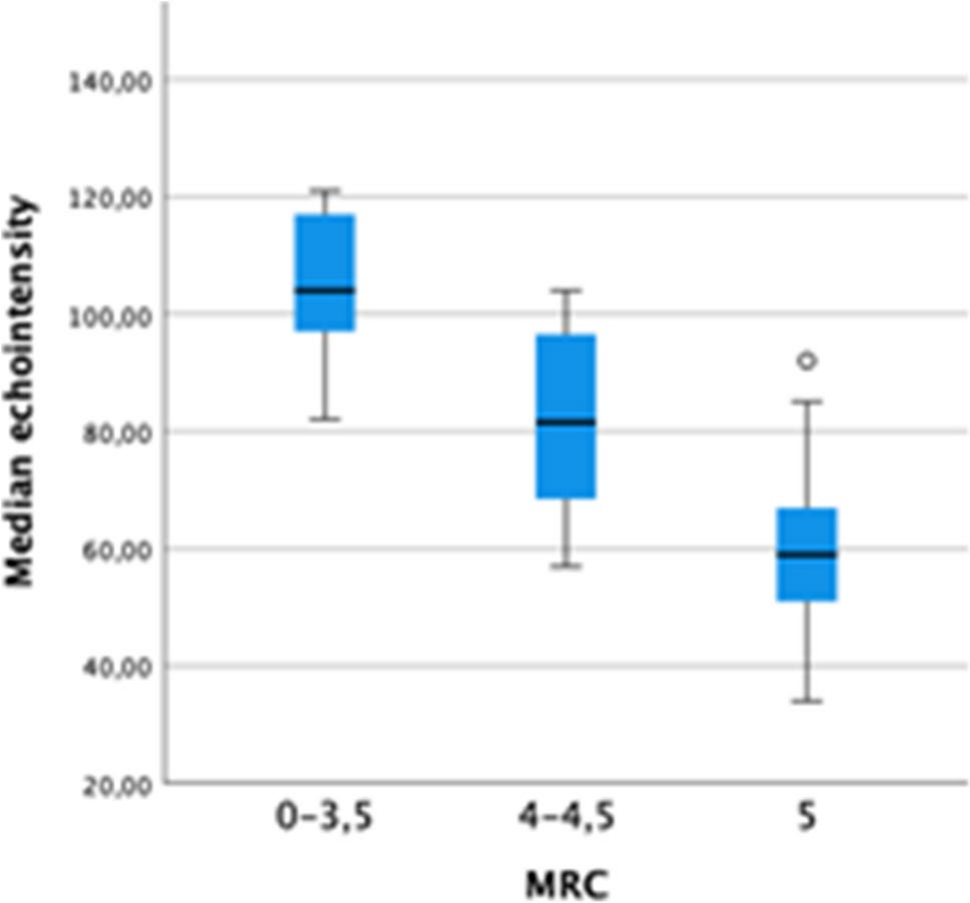
Inverse correlation between MRC sum score of *tibialis anterior* and median EI of the same muscle

To assess the relationship between EI and muscle strength as evaluated by MRC, we analysed the *tibialis anterior*, where we were able to observe a significant inverse correlation between MRC and median EI (*p* < 0.001) (Fig. 1).

### QMUS vs. muscle MRI

Out of the total number of 130 muscles scanned in FSHD patients with US, 111 were evaluable at MRI. Seventy-one (64%) muscles showed MRI changes, which consisted in fat replacement of grade 1 in 20 (18%), grade 2 in 13 (11.7%), grade 3 in 15 (13.5%) and grade 4 in 23 (20.7%) as per Mercuri grading scale.

Median EI was significantly higher in muscles with MRI changes than in muscles without MRI changes (*p* < 0.001). EI was similar in muscles with and without STIR hyperintensity. Nineteen muscles (17.1%) showed STIR hypertintensity and all muscles with STIR hyperintensity also showed T1 hyperintensity (Table 3).

**Table 3:** Comparison between muscle ultrasound and MRI data in 111 FSHD1 muscles

Muscle US	Muscle MRI						
Echointensity	T1* Hyperintensity				STIR Hyperintensity		
	Mercuri 0** *N* = 40	Mercuri 1–2** *N* = 33	Mercuri 3–4** *N* = 38	p	Absent *N* = 92	Present *N* = 19	p
Median	61.0	79.0	95.0	< 0.001	72.0	89.0	0.092
IQR	50.25–71.75	53.0–94.0	72.0–115.0		56.0–94.0	56.5–107.0	

^*^Overall T1 hyperintense muscles vs. Mercuri 0 muscles: *p* < 0.001. **Bonferroni correction for multiple tests: Mercuri 0 vs. Mercuri 1–2 *p* = 0.003; Mercuri 0 vs. Mercuri 3–4 *p* < 0.001; Mercuri 1–2 vs. Mercuri 3–4 *p* < 0.001

**Fig. 2:**
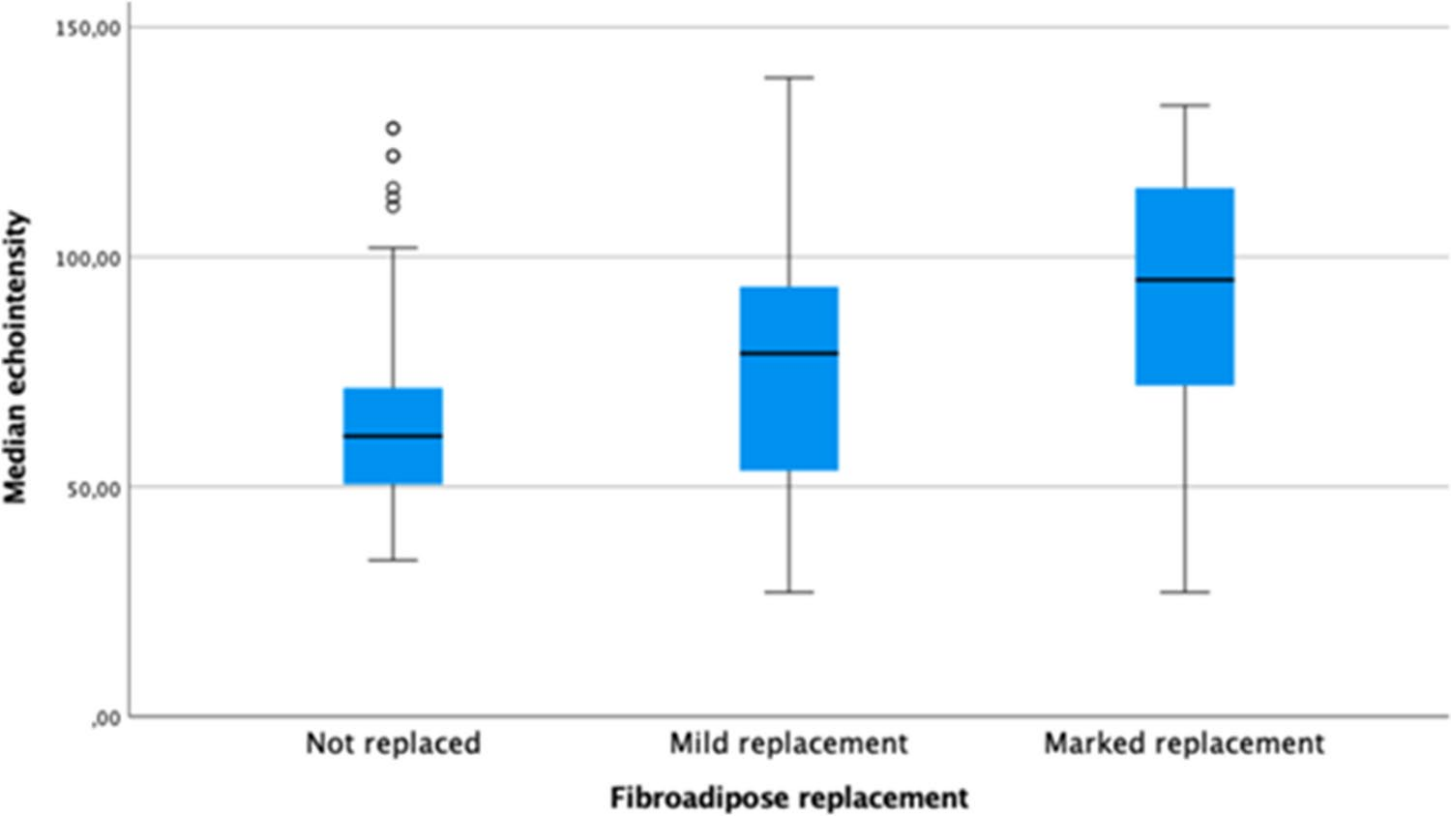
Median echointensity correlates with fat replacement divided into three categories: no replacement, mild replacement (Mercuri 1–2) and marked replacement (Mercuri 3–4)

Based on the grade of fat replacement, we considered three different MRI categories: no replacement, mild replacement (Mercuri 1–2) and marked replacement (Mercuri 3–4), and found a higher median EI in muscles with more severe fat replacement (*p* < 0.001) (Fig. 2). Pairwise comparison of muscle median EI with respect to fat replacement showed that median EI was significantly different in all three categories (no replacement vs. mild replacement *p* = 0.003; no replacement vs. marked replacement *p* < 0.001; mild replacement vs. marked replacement *p* < 0.001).

## Discussion

QMUS is able to quantify muscle EI by computer-assisted grey-scale analysis, which offers a high sensitivity and reliability in the detection of muscle alterations in numerous NMDs [24, 25]. In particular, QMUS demonstrated an overall detection rate of more than 90% in NMDs [26]. Furthermore, QMUS may be a sensitive measure able to quantify changes of muscle echogenicity over time, thus being also useful for follow-up evaluations [27, 28]. On these bases, QMUS, as MRI, may represent an imaging biomarker in muscular disorders [29].

In this study, we used FSHD as a pathological model to evaluate the contribution of QMUS in muscular imaging and its possible role with respect to MRI. By means of QMUS, we found in 13 FSHD patients a significant increase of muscle EI with respect to healthy controls, matched for age, gender and BMI. The statistical analysis showed that EI increase was independently associated with older age (both in patients and controls) and with disease severity, as evaluated by FSHD score, showing that QMUS can reflect muscle changes due to ageing, disease progression and disease severity. Accordingly, EI of *tibialis anterior* also correlated with muscle strength, as evaluated by MRC, confirming the role of a quantitative technique as outcome measures [30, 31].

**Fig. 3:**
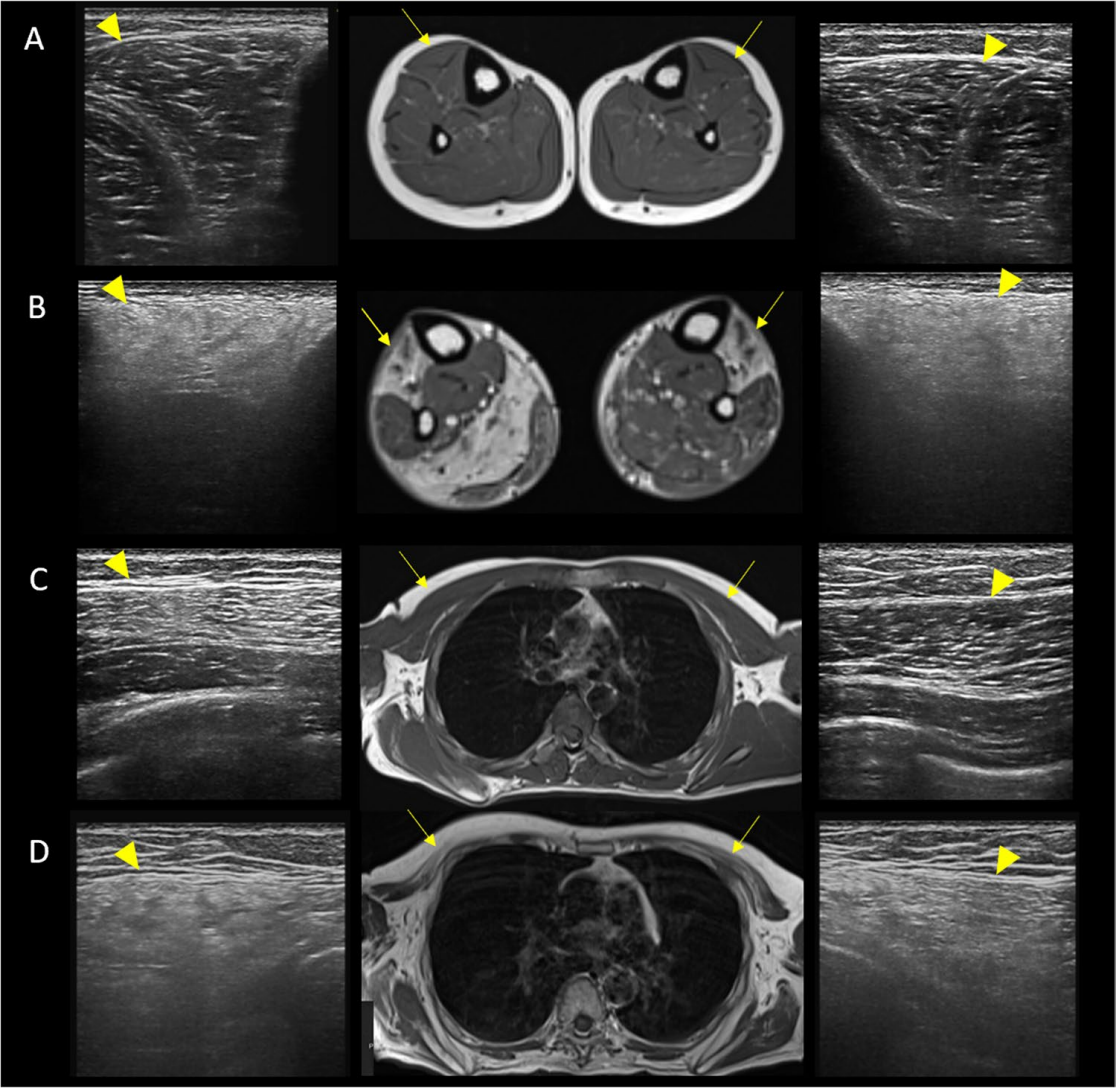
Muscle MRI and US matching. **A** Images of right and left tibialis anterior, appearing normal on MRI (Mercuri scale 0) (yellow arrows) and on US (yellow arrowheads). **B** Images of right and left tibialis anterior, completely fat replaced at MRI (Mercuri scale 4) (yellow arrows), and the corresponding US image that shows increased echogenicity and structural tissue changes (yellow arrowheads). **C** Images of right and left pectoralis major, appearing normal on MRI (Mercuri scale 0) (yellow arrows), while the right US image shows increased echogenicity and the left US image shows normal muscle echogenicity. **D** Images of right and left pectoralis major, appearing fat replaced at MRI (right: Mercuri scale 3, left: Mercuri scale 2) (yellow arrows), and the corresponding US image that shows increased echogenicity and structural tissue changes, more pronounced in the right muscle (yellow arrowheads)

In our study, EI was increased in all FSHD muscles, while only 64% of them showed qualitative MRI changes (Mercuri 1–4). In particular, QMUS was able to identify muscle alterations also in the presence of mild or no MRI changes (Fig. 3), demonstrating a good sensitivity of QMUS, in agreement to what was reported in a previous study [29]. It has been highlighted the delay between histopathological abnormalities and the onset of muscle MRI alterations in some myopathies [32]; it could be hypothesized that, especially in superficial muscles where US has the best resolution, QMUS could identify the presence of fat replacement in advance with respect to MRI. Interestingly, QMUS showed significant increase of EI in *deltoid* muscle, which is generally spared in FSHD imaging. In our study, scanned muscles were chosen based on both the general knowledge of muscle involvement in FSHD [16] and the accessibility of the single muscles to US examination. Both the muscle selection that limited the study to five superficial muscles and the semi-quantitative nature of the analysis of T1-weighted MRI images may have influenced the different sensitivity of QMUS vs. MRI. However, this is an important point because, while the availability and applicability of muscle MRI is limited in many centres, muscle US is a fast and immediate technique which, simply by setting a specific program on a common US machine, makes it possible to collect quantitative measurements and to provide important clinical and diagnostic information.

QMUS and MRI data in the single muscles showed that the most T1-replaced muscles were the most hyperechoic (Fig. 3). However, we were unable to find any correlation between STIR hyperintensity and muscle echogenicity. STIR hyperintense muscles usually mean the presence of edema [33], which should make muscle appear hypoechoic on US. However, we observed that all muscles with STIR hyperintensity showed also T1 alterations and increased EI, pointing out a limit of US technique: muscle edema and fat replacement can coexist, and in this case, their distinction is possible with MRI, but not with US.

Accordingly, QMUS and semiquantitative MRI should be considered complementary techniques with different advantages: MRI can better analyse different types of morphological alterations (e.g. edema vs. fat replacement), is able to evaluate deeper muscles and is not affected by the presence of a thick adipose panniculus; on the other hand, QMUS is a dynamic assessment of muscular echogenicity, which allows to study different muscles in their whole length within a reasonable time and lower costs; it has a good sensitivity for fat replacement, with an excellent reliability.

Some limitations in our study deserve to be highlighted. The main ones are represented by the small size of our cohort and the heterogeneity of the sample in terms of disease progression and muscle impairment. Additional studies with wider samples are needed to determine the accuracy of QMUS and its sensitivity also in longitudinal studies. To date, in neuroimaging studies in NMDs, quantitative MRI methods are increasingly used to assess muscle alterations, as they are more objective and sensitive tools [31, 34]. However, in this study, we compared QMUS data with semi-quantitative MRI scales as they are the most widely available and commonly used in clinical practice.

## Conclusions

In our study, we demonstrated that QMUS has a good sensitivity in detecting structural muscular alterations in FSHD, supporting its potential use as a screening and diagnostic tool in NMDs in general. Furthermore, its good repeatability confirms the potential applicability of this technique in follow-up studies and in the context of clinical trials as well [27, 28]. In particular, while qualitative measures require an experienced observer for interpretation (a role that in a trial setting should be centralized), QMUS offers the possibility to collect homogeneous data on muscle alterations and compare them with control populations in multicentre studies. Finally, a few aspects in the practical application of this tool are worth mentioning: it can be performed at bedside, is less expensive than MRI, can evaluate different limb muscles in a short span of time and could come in handy as a useful tool for US-guided biopsies.

## Data Availability

Original supporting data are electronically stored at our Institution are available on request.
